# Genotypic variations and clinical implications of JEV-associated peripheral nerve injury: a commentary on multicenter findings from high-endemic regions

**DOI:** 10.1080/22221751.2024.2449073

**Published:** 2025-01-09

**Authors:** Liangping Zhang, Lei Pan, Rongqi Cao

**Affiliations:** Department of Orthopaedics, Zhejiang Chinese Medical University, Hangzhou, People’s Republic of China

**Keywords:** Japanese encephalitis, nerve injury, genotype, vaccination, early diagnosis

## Abstract

We read with great interest the recent article by Wang et al. on peripheral nerve injury (PNI) associated with Japanese encephalitis virus (JEV) infection in high-endemic regions of China. The study provides important insights into the significant relationship between JEV infection and PNI, particularly highlighting clinical manifestations such as acute flaccid paralysis and respiratory muscle paralysis. While we commend the authors' work, we suggest caution in interpreting the findings due to several limitations. First, genotype-specific differences, notably between GIb and GIII strains, may influence disease severity, clinical progression, and prognosis, warranting further investigation for personalized management. Second, although adjustments were made for certain demographic and epidemiological variables, additional confounders such as vaccination status, environmental conditions, and socioeconomic factors should be incorporated to strengthen the robustness of future analyses. Third, reliance on surveillance data introduces potential biases due to incomplete or inaccurate reporting, especially in rural or underserved populations. Enhanced data collection methods, including digital health tools and standardized questionnaires, could improve accuracy and comprehensiveness. Beyond methodological considerations, the study underscores the importance of early diagnosis, biomarker development, and multidisciplinary collaboration in mitigating neurological complications of JEV. Strengthening vaccination coverage, particularly in remote regions, and expanding health education are also critical to reducing disease burden. Overall, this research advances understanding of JEV-associated PNI and highlights avenues for future studies to refine diagnostic, preventive, and therapeutic strategies that will improve long-term patient outcomes.

To the Editor:

We were very interested in reading the recent publication by Guowei Wang et al. in Emerging Microbes & Infections, titled “Peripheral nerve injury associated with JEV infection in high endemic regions, 2016–2020: a multicenter retrospective study in China [1].” As healthcare professionals, we are pleased to see the authors’ focus on peripheral nerve injury (PNI) related to Japanese encephalitis virus (JEV) infection. This study highlights a significant association between JEV infection and peripheral nerve injuries, such as acute flaccid paralysis and respiratory muscle paralysis, in high-endemic areas along the Yellow River in China, particularly offering a stratified analysis of patient types with critical clinical insights. We appreciate the authors’ work, which significantly contributes to our understanding of the relationship between JEV infection and nerve injury, though the results should be interpreted with caution for several reasons.

Firstly, regarding the subgroup analysis of JEV genotypes, such as GIb and GIII, we believe that genotype differences may have significant implications for clinical manifestations and disease progression. Different genotypes of JEV may exhibit variations in epidemiological characteristics and clinical presentations of neurological damage. For instance, patients infected with the GIb genotype tend to exhibit more severe clinical symptoms, such as acute flaccid paralysis and respiratory muscle paralysis, whereas those infected with the GIII genotype generally experience milder nerve damage and have a better prognosis. Future research could focus on detailed analyses of the clinical features associated with different genotypes to better understand their impact on disease progression and treatment response. This would aid in the development of more personalized treatment strategies for patients infected with different genotypes.

Secondly, confounding variables. Although the study adjusted for variables such as patient age, sex, and medical history, and accounted for epidemiological characteristics of different regions and seasons, other variables, including vaccination status (e.g. unvaccinated or waning immunity post-vaccination), environmental factors (e.g. climate, mosquito density, living conditions), and socioeconomic factors (e.g. healthcare access and sanitation), could further strengthen the robustness of the results [2]. These additional variables may play critical roles in the disease progression and treatment outcomes.

Thirdly, the study was based on case data obtained from the Chinese Center for Disease Control and Prevention (CDC) surveillance system, collecting information on JEV infection and neurological outcomes. The limitations of these data are evident, as they were primarily evaluated based on laboratory tests and clinical reports from hospitals. Patients from remote areas or with lower educational levels may not provide accurate self-reports of medical history and symptom progression. Observational data on neurological impairments and treatment responses may also be incomplete or inaccurate. This could impact the accuracy and reliability of the study results. Future research should incorporate more advanced health monitoring tools, such as smartphone applications, wearable devices, and standardized questionnaires, to ensure more precise and comprehensive patient data collection.

Despite the authors’ suggestions, some introspection is essential for us as healthcare professionals. Firstly, this study highlights the significant relationship between Japanese encephalitis virus (JEV) infection and peripheral nerve injury (PNI), underscoring the critical importance of early diagnosis and intervention, particularly in high-endemic regions. Future research may focus on exploring neurological injury biomarkers to screen high-risk populations, thereby enhancing early detection and diagnostic accuracy to improve patient prognosis. Secondly, the article emphasizes the crucial role of multicenter collaboration and large-scale data collection in understanding JEV-associated neurological injuries. Infectious disease specialists, neurologists, critical care physicians, and rehabilitation experts play vital roles in multidisciplinary care. Such collaboration not only helps elucidate the diverse effects of JEV infection on the nervous system but also facilitates the dissemination of optimal treatment strategies. Additionally, in remote areas with low vaccination coverage, strengthening public health policies and expanding vaccine promotion efforts are essential to reducing JEV transmission rates and the incidence of severe neurological injuries. Finally, healthcare professionals can leverage their expertise to advocate for stronger vaccination programs and health education initiatives while providing patients with long-term rehabilitative support. Combining early detection, vaccination, and personalized rehabilitation not only reduces the occurrence of severe cases but also enhances the overall quality of life for patients.

In conclusion, we commend this article for its focus on the association between JEV infection and peripheral nerve injury, as well as its observations on different neurological injury patterns. We advocate for early identification and intervention in high-risk populations with personalized prevention and treatment approaches. Future research should build on these findings to develop better diagnostic tools and more effective interventions to further improve long-term patient outcomes.
